# Survey of antiretroviral therapy adherence and predictors of poor adherence among HIV patients in a tertiary institution in Nigeria

**DOI:** 10.11604/pamj.2019.33.277.18711

**Published:** 2019-07-31

**Authors:** Adekunle Olatayo Adeoti, Mobolaji Dada, Tobiloba Elebiyo, Joseph Fadare, Opeyemi Ojo

**Affiliations:** 1 Department of Medicine, Ekiti State University Teaching Hospital, Ado-Ekiti, Ekiti State, Nigeria; 2 Department of Psychiatry, Ekiti State University Teaching Hospital, Ado-Ekiti, Ekiti State, Nigeria; 3 Department of Biochemistry, University of Ibadan, Oyo State, Nigeria

**Keywords:** Adherence, predictors, HIV/AIDS, CD4 count, mental health

## Abstract

**Introduction:**

Adherence is vital to effective antiretroviral therapy (ART) for reducing viral load and HIV/AIDS-related morbidity and mortality. This study was aimed at evaluating the adherence of HIV seropositive patients to ART in a tertiary institution in Nigeria.

**Methods:**

A cross sectional observational study was conducted among 400 HIV seropositive patients. The study was carried out between December 2016 and February 2017 at the HIV clinic of the Ekiti State University Teaching Hospital, Ado-Ekiti, Nigeria.

**Results:**

The mean age of the HIV patients was 42.2±9.5 years with a predominant female gender (Male:Female = 1:2.8). The median CD4 counts increased from 302.1±15.0cells/mm^3^ at diagnosis to 430.8±13.3cells/mm^3^ at the time of the study. Majority of participants were unaware of their spouses' HIV status (59.3%) while 32.5% of participants had a serodiscordant spouse. Poverty was a major challenge as 73.3% earned less than 140 dollars per month. Depressive symptoms, anxiety disorder and insomnia were also reported in 40.7%, 33.2% and 47.2% respectively. Poor adherence to ART was observed in almost 20% of the patients. Logistic regression indicated that predictors of poor adherence were depression, anxiety and low CD4 counts.

**Conclusion:**

Adherence to anti-retroviral therapy was good amongst the majority of HIV seropositive patients. Depression, anxiety disorder and low CD4 count were however associated with poor adherence. This emphasizes the role of the psychology units as integral part of the HIV clinic to assist patients' adherence to anti-retroviral regimens.

## Introduction

HIV remains a major pandemic that has claimed more than 35 million lives over the last three decades [1]. In 2017, approximately a million HIV related death and 1.8 million newly infected were reported by the World Health Organization with a major burden in sub-Saharan Africa [2]. Globally, nearly 40 million people are living with the disease, with a decline in the annual death from AIDS-related causes by nearly half in the past ten years, but this is still higher than the proposed UN target of 500,000 deaths in 2020 [3]. Since the first two AIDS cases were diagnosed in Nigeria in 1985, there has been an increase in the number of new cases with 9% of the global HIV burden coming from this region [4]. Antiretroviral therapy (ART) transformed this potentially incurable disease to a manageable chronic illness by suppressing the viral load and reducing the risk of transmission of the disease [5, 6]. Nevertheless, the continued success of ART is highly dependent on early initiation of therapy, continuity in care and high treatment adherence [7]. Drug adherence is the key factor in disease control, as ART adherence of ≥95.0% can achieve suppression of viral load to undetectable levels, improve immune system function and reduce AIDS-related morbidity and mortality [8, 9]. In addition, achieving the UNAIDS 90-90-90 targets (90% of all people living with HIV will know their HIV status, 90% of those people will be on ART and 90% of them will be virally suppressed) issues of adherence to ART and viral suppression need to be given more attention [10]. An observational study indicated that just 62% of HIV infected patients take at least 90% of their prescribed ART doses [11]. Therefore, drug adherence is a major challenge to effective ART patient management and the development of ART adherence intervention research is crucial for effective HIV management with the aim of achieving the 90-90-90 goal of the UNAIDS/WHO [7]. This study was aimed at evaluating the adherence of HIV seropositive patients to ART treatment regimens in a tertiary institution in Nigeria.

## Methods

**Study design and setting:** this study was a cross sectional observational study which was carried out at the HIV clinic of Ekiti State University Teaching Hospital (EKSUTH), Ado-Ekiti, Nigeria between December 2016 and February 2017. This HIV clinic runs in conjunction with Ekiti State Agency for Control for HIV/AIDS and the implementing partners under the support of the Federal Ministry of Health, Nigeria. EKSUTH is the major tertiary institution in the state capital serving a population of over 2 million people in the state. In 2014, the sentinel HIV survey reported the prevalence of HIV/AIDS in Ekiti State as 2.9%, which was one of the lowest prevalence in the country [12].

**Sampling and sample size:** Raosoft incorporation software was used to calculate the sample size of 327 using a 95% confidence level [13]. Furthermore, this was increased to 400 for absolute precision of 5% points to have sufficient variation in the population characteristics (age and sex) that may influence adherence as well as power of the study. The list of 1,128 adult HIV patients from HIV clinic register was used to select 400 patients by simple random sampling method. Patients who were aged 18 years or older and had been on anti-retroviral therapy for a minimum period of 6 months were recruited for the study. Patients below 18 years, drug addicts, patients on chronic use of other medications aside anti-retroviral drugs or patients whose treatments were interrupted due to adverse effects were excluded from the study.

**Data collection tools:** the modified 8-item Morisky Medication Adherence Scale (MMAS-8), a validated self-reported questionnaire, was used to assess the adherence of patients to medication [14]. It had eight questions to assess the knowledge and motivation levels of participants regarding adherence. Each question was used in determining a specific type of adherence behaviour. In order for a participant to achieve an optimum result, seven of the questions must have had a negative response while one of them must have had a positive response. One of the questions was answered using a scale of five options: never; almost never; sometimes; often and always. Scoring was graded as high adherence (0 points); medium adherence (1 and 2 points); poor adherence (above 2 points).

A closed ended self-assessment questionnaire was employed in the collection of data regarding number of medications which have been taken, number of doses missed by each of the participants, socio-demographic information, information regarding family support and reasons for not taking medications as directed by their doctor. In addition, the Hospital Anxiety Depression Scale (HADS) was also used to assess the prevalence of anxiety and depression among the respondents [15]. Insomnia among the patients was assessed with insomnia severity index scale. The questionnaires were administered to consenting patients who met the inclusion criteria [16]. All recruited patients were on same antiretroviral therapy combination (Atripla-Efavirenz 600mg/Emtricitabine 200mg/Tenofovir 300mg) for at least six months. The lymphocyte CD4 cell counts were measured for eligible patients who had been on ART for the stipulated time period using flow cytometer (Cyflow Partec Counter 2) likewise the viral load was determined using polymerase chain reaction.

**Data analysis:** data collected were analyzed using SPSS 20. Categorical variables were expressed in proportions and compared using chi-square while continuous variable were expressed in means and compared using T-test. In order to explore the factors associated with lower adherences scores, a logistic regression analysis was employed. P-value <0.05 will be considered as statistically significant. For ease of analysis, the adherence categories comprising of low, medium and high adherence were merged into two groups. The medium and high adherences were merged together as “good adherence” while low adherence remained as “poor adherence”.

**Ethical approval:** approval was obtained from the Ethical and Research committee of Ekiti State University Teaching Hospital, Ado-Ekiti. Informed consent was obtained from all participants and confidentiality was ensured in the obtained information.

## Results

**Socio-demographic characteristics of HIV patients:** four hundred HIV seropositive patients were randomly selected for the study. The socio-demographics of the patients presented in Table 1, indicated that their mean age was 42.18±9.5 years with almost three quarter of the respondents being females (74%). A large proportion of the participants were married (76%) and self-employed (60.7%). Most of the patients had formal education as a negligible percentage of 1% of the participants had no formal education. It was observed from the study that a large proportion of the patients were low income earners with monthly pay below 140 dollars (less than 5 dollars per day). The majority of the HIV infected patients were unaware of their partners HIV status (59.3%) while 32.5% had serodiscordant spouse.

**Table 1:** Socio-demographics of the HIV patients

Variable	Frequency	Percentage (%)
**Age in years (42.2 ± 9.5 years)**		
Below 30	31	7.8
30-50	284	71.0
Above 50	85	21.2
**Sex**		
Male	104	26.0
Female	296	74.0
**Marital status**		
Single	42	10.5
Married	304	76.0
Divorced	24	6.0
Widowed	30	7.5
**Education**		
None	4	1.0
Primary	68	16.5
Secondary	144	34.9
Tertiary	197	47.7
**Average income per month**		
< 140 dollars	289	73.3
140–280 dollars	86	23.4
> 280 dollars	12	3.3
**HIV Status of spouse/partners**		
Unknown	237	59.3
Positive	33	8.2
Negative	130	32.5

Exchange rate at 357 Naira to 1 dollar

Values are expressed in median ± SD

**Clinical characteristics:** the mean CD4 count of the HIV seropositive patients at diagnosis was an average of 302.06±15.82cells/mm^3^ (mean ± SEM). It also showed that about half of the HIV infected patients (44.8%) had an initial CD4 count below 200cells/mm^3^. An increase in the CD4 count of the patients to an average of 430.8±13.30 cells/mm^3^ (mean ± SEM) was observed, with a high proportion of the seropositive patients (34.9%) having a CD4 count above 500 cells/mm^3^ at the time of the study and also the viral load of half of the patients (51.3%) was undetectable (<50 copies). About forty percent of the patients were clinically depressed, 47.2% had insomnia and 33.2% had anxiety disorder while 81% had good adherence to their medications as shown in Table 2.

**Table 2:** Clinical characteristics of the HIV patients

Variables	Frequency	Percentage (%)
**Initial CD4 (cells/mm^3^)**		
Below 200	179	44.9
200-350	89	22.3
351-500	68	17
Above 500	63	15.8
**Current CD4 (cells/mm^3^)**		
Below 200	70	17.8
200-350	81	20.6
351-500	105	26.7
Above 500	137	34.9
**Viral load (number of copies)**		
Below 50 (undetectable)	98	51.3
50 -1000	61	31.9
Above 1000	32	16.8
**Depression (Median depression score = 7 ± 4.4)**		
Non case	273	59.3
Depression	163	40.7
**Insomnia (Median insomnia index = 6 ± 5.5)**		
Clinically insignificant	211	52.8
Insomnia	189	47.2
**Anxiety (6 ± 3.9)**		
Non case	267	66.8
Anxiety	132	33.2
**Adherence (1 ± 1.2)**		
Poor	78	19
Good	323	81

Values are expressed in median ± SD

P is statistically significant at p< 0.05

**ART adherence and associated factors:** multivariate analysis on the association of ART adherence against predictors such as; gender, age, CD4 count, viral load, insomnia index, depression score and anxiety score were carried out using binary logistic regression. Table 3 shows that the predictors of poor adherence were depression, anxiety and CD4 count below 200 cells/mm^3^. As presented in Table 3, the odds of depressed patients adhering to ART regimens were 0.2 times lower than patients who were not depressed (0.001). The result also indicates that seropositive patients suffering from anxiety are less adherent to ART (0.25 times, p=0.006). The current CD4 count of the patients was also a predicator of non-adherence as seropositive patients with CD4 below 200 were 15 times less adherent to ART regimens in comparison to patients whose CD4 count was between 200-350 cells/m^2^ (p=0.0005). As indicated by the results presented in the table, patients with CD4 below 200 cells/m^2^ also had ART adherence levels that were 3.75 times lower than those of patients with CD4 above 500 cells/m^2^ (p=0.001).

**Table 3:** Logistic regression analysis showing associations between adherence and potential predictors

Variables	Adherence		OR (95% Cl)	p-value
	**Good n (%)**	**Poor n (%)**		
**Age**				
Below 30	23 (67.6)	11 (34.4)	1	0.991
30 -50	230 (80.1)	57 (19.9)	1.09 (0.18 – 6.73)	0.925
Above 50	71 (91)	7 (9)	0.99 (0.30 – 3.22)	0.985
**Sex**				
Male	78 (74.3)	27 (25.7)	1	0.050
Female	246 (83.7)	48 (16.3)	2.64 (0.99 – 6.96)	0.050
**Marital status**				
Single	27 (62.8)	16 (37.2)	1	0.101
Married	245 (81.7)	55 (18.3)	2.47 (0.34 – 18.09)	0.374
Divorced	24 (96)	1 (4)	0.87 (0.16 – 4.58)	0.865
Widowed	28 (90.3)	3 (9.7)	0.09 (0.01 – 1.48)	0.092
**Depression**				
Not depressed	205 (86.9)	31 (13.1)	1	0.001
Depressed	119 (73)	44 (27)	0.20 (0.08 – 0.52)	0.001
**Insomnia**				
Absent	177 (70)	33 (30)	1	0.561
Present	147 (77.8)	42 (22.2)	0.78 (0.34 – 1.78)	0.561
**Anxiety**				
Absent	235 (82.7)	49 (17.3)	1	0.006
Present	89 (78.1)	25 (21.9)	0.25 (0.09 – 0.67)	0.006
**Viral load**				
Suppressed	303 (81.7)	68 (18.3)	1	0.163
Unsuppressed	21 (75)	7 (25)	0.36 (0.09 – 1.58)	0.163
**CD4 at diagnosis**				
Below 200	133 (79.6)	34 (20.4)	1	0.147
200-350	67 (80.7)	16 (19.3)	0.29 ( 0.08 – 1.03)	0.056
351-500	54 (85.7)	9 (14.3)	0.97 (0.28 – 3.37)	0.963
Above 500	54 (84.4)	10 (15.6)	0.56 (0.15 – 2.09)	0.388
**Current CD4**				
Below 200	37 (57.8)	27 (42.2)	1	0.0005
200-350	68 (91.9)	6 (8.1)	15.15 (4.24 – 54.04)	0.0005
351-500	82 (81.2)	19 (18.8)	1.45 (0.38 – 5.58)	0.592
Above 500	133 (86.9)	17 (13.7)	3.75 (1.38 – 10.19)	0.001

P is statistically significant at p< 0.05

## Discussion

In this study, majority of the HIV seropositive patients were young females in their reproductive age. Nigeria has the second largest HIV epidemic in the world [17]. It is estimated that 58% of people living with HIV and AIDS in Nigeria are women [18]. The underlying reason why a larger proportion of women are infected with HIV in comparison to men in Nigeria has been attributed to deep roots of gender inequality in Nigeria society, culture and law [19]. The predominantly young, female population in our study could be due to more sexually active among this younger age group and the receptive sexual anatomic design in females [20]. On the contrary, in countries with higher prevalence of HIV infection amongst homosexual men, more males were reported to be HIV positive [21]. A preponderance of the patients showed good adherence to their ART, however the minority with poor adherence had associations with depression, anxiety disorder and low CD4 count. Good adherence to ART has revolutionized HIV medicine as it leads to suppressed viral load and repopulation of diminished CD4 T-lymphocytes and the resultant decreases mortality as well as risk of opportunistic infection [22]. Our study shows an increase in the average CD4 count from diagnosis to the time of commencement of this study. There was a significant increase in the CD4 count of the seropositive patients. Also, a high proportion of the seropositive patients had undetectable viral load which indicates minimized risk of transmitting HIV infection to their partners, by viral load suppression, as a third of the respondents had serodiscordant sexual partners [23].

Similar to other studies, significant percentage of the HIV seropositive patients in our study had depression, anxiety disorder and insomnia [24, 25]. These co-existing manifestations have also been reported as sleep disturbances was said to be prevalent amongst the patients with underlying depression [26]. Furthermore, anxiety could be associated to the thought of the illness, fear of the future and financial concerns, as the majority of the study participants earned less than 140 dollars per month [27]. Most of the HIV seropositive patients were adherent to anti-retroviral therapy which was similar to other studies in sub-Saharan Africa with pooled estimate of 77% adherence but higher than adherence of 55% in North America [28]. This discrepancy could be due to the effectiveness of the HIV programme in this region and the free HIV treatment funded by the partners, which most patients could consider as a new opportunity to live. In our study, predictors of poor adherence to ART drugs were depression, anxiety disorder and low CD4 count which have been reported in similar studies [29, 30]. Unidentified mental health disorders among people living with HIV may prevent the actualization of the last 90 in the UNAIDS-90-90-90-target. This has significant impact on their quality of life, disease progression and mortality [30]. Thus, regular and active psychological assessment for all HIV patients becomes imperative for a holistic care in order to achieve this target. Poor adherence, accompanying sub-optimal antiretroviral effect of medication and its attendant development of drug resistant strains of HIV, low CD4 count and unsuppressed viral load could be responsible for the low CD4 count as a predictor in our study [31, 32]. Furthermore, the majority of the patients in the study presented in advanced stage of the disease with low CD4 count. This late presentation could account for slower increase in CD4 cell count especially in patients with virological failure [29].

**Limitation of the study:** a multi-center study and community based study would have been more representative compared to our study which was hospital based. Also, the data on adherence was based on responses from patients which may have some level of bias.

## Conclusion

This study indicates a good adherence in majority of the patients but a small proportion with predictors of poor adherence to anti-retroviral therapy which were depression, anxiety disorder and low CD4 count. An integration of a psychology unit in the HIV programme with regular and active monitoring of patients will be crucial for improved adherence.

### What is known about this topic

- Adherence to antiretroviral medication is crucial to medication effectiveness;
- Viral load suppression is necessary in achieving WHO/UNAIDS 90-90-90 goal;
- Drug adherence reduces the risk of the development of resistant strains.

### What this study adds

- The predictors of poor antiretroviral adherence in Ekiti State University Teaching Hospital (EKSUTH);
- Identified the key role of mental health as an integral component of HIV medicine;
- Estimated the level of adherence of HIV seropositive patients in EKSUTH.

## Competing interests

The authors declare no competing interests.
